# Linkage disequilibrium mapping via cladistic analysis of phase-unknown genotypes and inferred haplotypes in the Genetic Analysis Workshop 14 simulated data

**DOI:** 10.1186/1471-2156-6-S1-S100

**Published:** 2005-12-30

**Authors:** Caroline Durrant, Andrew P Morris

**Affiliations:** 1 Wellcome Trust Centre for Human Genetics, University of Oxford, Roosevelt Drive, Headington, Oxford, UK

## Abstract

We recently described a method for linkage disequilibrium (LD) mapping, using cladistic analysis of phased single-nucleotide polymorphism (SNP) haplotypes in a logistic regression framework. However, haplotypes are often not available and cannot be deduced with certainty from the unphased genotypes. One possible two-stage approach is to infer the phase of multilocus genotype data and analyze the resulting haplotypes as if known. Here, haplotypes are inferred using the expectation-maximization (EM) algorithm and the best-guess phase assignment for each individual analyzed. However, inferring haplotypes from phase-unknown data is prone to error and this should be taken into account in the subsequent analysis. An alternative approach is to analyze the phase-unknown multilocus genotypes themselves. Here we present a generalization of the method for phase-known haplotype data to the case of unphased SNP genotypes. Our approach is designed for high-density SNP data, so we opted to analyze the simulated dataset. The marker spacing in the initial screen was too large for our method to be effective, so we used the answers provided to request further data in regions around the disease loci and in null regions. Power to detect the disease loci, accuracy in localizing the true site of the locus, and false-positive error rates are reported for the inferred-haplotype and unphased genotype methods. For this data, analyzing inferred haplotypes outperforms analysis of genotypes. As expected, our results suggest that when there is little or no LD between a disease locus and the flanking region, there will be no chance of detecting it unless the disease variant itself is genotyped.

## Background

Disease-marker association studies of samples of unrelated cases and controls have been shown to have the potential to map all but extremely rare variants contributing to complex traits [1]. We recently described a method [2] for linkage disequilibrium (LD) mapping, using cladistic analysis of single-nucleotide polymorphism (SNP) haplotypes in a logistic regression framework, which allows straightforward incorporation of covariates. Under the assumption of multiplicative disease risks, the model is parameterized in terms of the haplotypic log odds of disease, although the form of the linear predictor can be generalized to other disease models. Cladistic analyses take advantage of the expectation that 'similar' haplotypes in the region flanking a disease locus tend to have similar risks of disease. Thus, by grouping haplotypes according to their similarity in terms of the alleles they carry, we expect to have greater power to detect a susceptibility locus for complex disease with a high-risk clade of haplotypes than with the individual haplotypes themselves [2].

In general, haplotype phase is not known and not deducible in samples of unrelated individuals. Determining haplotypes experimentally is too costly to consider for the large samples that will be needed to detect variants contributing to complex disease. Alternatively, a number of statistical approaches have been developed to infer haplotypes and their relative frequencies in a sample and to assign phase to the multilocus genotypes. Here we use a simple expectation-maximization (EM) algorithm to infer haplotypes, in the interests of speed for large datasets. It is common to employ a two-stage approach of inferring phase and then analyzing the 'best' haplotype configuration as if it were known with certainty. The disadvantage of this approach is that we cannot take account of the uncertainty in the phase assignment process. To overcome this problem, we propose in this paper a generalization of our cladistic analysis method for haplotypes to analyze unphased genotypes directly. We use the Genetic Analysis Workshop 14 (GAW14) simulated dataset to compare the analysis of unphased and inferred haplotype analysis in terms of power and accuracy to locate disease loci.

## Methods

### Cladistic analysis of haplotypes

Large regions are analyzed using a sliding window of SNPs, with separate analyses performed in each window. Phase is estimated in each window independently using the EM algorithm and for every individual, the resulting best-guess haplotype pair is included in the analysis as if it was known to be correct, regardless of its posterior probability. The pair-wise diversity of the resulting set of haplotypes is based on the number of marker matches within the window. Ignoring disease status, group-average hierarchical clustering techniques are used to construct a dendogram of distinct haplotypes by grouping together increasingly dissimilar clusters until all haplotypes are combined into a single clade. Information on the disease status of the haplotypes is then included, and at each level of the dendogram a likelihood ratio test is calculated with the null hypothesis of a random distribution of 'affected' haplotypes across clusters. The most significant test statistic over all levels of the dendogram, T[MAX], and its exact *p*-value calculated from permutations of the case/control labels, are reported for each window.

### Analysis of unphased multilocus genotypes

In practice, phase-known data are generally not available, so we have generalized our method to analyze multilocus genotypes directly. The approach is directly analogous to that used to analyze phase-known haplotypes [2], but with different weighting used in the pair-wise distance measure. Consider a sample of individuals typed at M SNPs, where *g*_*im *_denotes the unphased genotype of individual *i *at locus *m*, coded as 11, 12, or 22 with 0 for missing data. Then the pair-wise distance measure is , such that



where *q*_*k[m] *_denotes the sample's relative frequency of genotype *k *at locus *m*. As with the weights used in the haplotype measure, this gives more weight to matching rare alleles at a marker than to matching common alleles.

Our method is designed for high-density SNP data, so we opted to analyze the GAW14 simulated dataset. The marker spacing in the initial screen was too large for our method to be effective, so we used the answers provided to select regions of interest to request further data. We chose ten regions of two adjacent packets of markers, containing up to 40 SNPs after the microsatellites were removed. These regions were chosen to cover the four main disease loci and the two modifier loci. In addition, we randomly selected four other regions not containing disease loci, or null regions, two with background LD between loci and two without, to check false-positive error rates. The four regions correspond to packet numbers 364–365, 120–121, 178–179 and 287–288.

Cases were selected as the affected founders from each family, with one case randomly selected from families with no affected founders, where affection status was determined from the phenotypes P1, P2, and P3. The four subpopulations of cases were analyzed separately to allow for any stratification because the subpopulation of the controls was unknown. For each subpopulation, all 100 replicates were analyzed individually to assess power, with each replicate dataset consisting of the selected cases and the controls for that replicate. Each region was analyzed using a sliding window of six markers, with separate analyses performed in each window. To allow for multiple testing, exact regional *p*-values were calculated for each region based on the best result seen across all windows within the region, using 100 random permutations of the case/control labels. Power was calculated as the proportion of replicate datasets with regional *p*-value less than 0.05. Accuracy of location was measured as the percentage of replicate datasets with a regional *p*-value less than 0.05 in which the most significant window in the region contained the disease locus.

## Results

### False-positive error rates

False-positive error rates were estimated over all four subpopulations for the two analyses by considering the four null regions, and were calculated as the proportion of data replicates with an exact regional *p*-value less than 0.05. Table 1 contains the mean false-positive error rates for regions with and without LD, with 95% confidence intervals, for the two analyses. The false-positive rates for both analyses (Table 1) are consistent with a 5% significance threshold, even in the presence of background LD in the region. The rate for the genotype analysis is slightly lower than the rate for the haplotype analysis regardless of whether there is background LD in the flanking region.

### Power

Table 2 contains the power and accuracy of location (as the percentage of regionally significant data replicates) of the two analyses to detect the six disease loci in the four subpopulations. There are no accuracy estimates for locus D2 because it is placed on the end of a chromosome and is not covered by a sliding window. In general, the power of the haplotype analysis is also greater than the power of the genotype analysis. The power of the likelihood ratio test, T[MAX], is affected by its degrees of freedom, which is determined by the number of different genotypes or haplotypes observed. Because there will usually be more multilocus genotypes than haplotypes in a sample, we expect the genotype analysis to be less powerful. The haplotype analysis also appears to have slightly greater accuracy than the genotype analysis in general, although not when there is little power to detect a locus in a population, when the accuracy varies considerably. This is due to the small number of results considered in relation to the number of windows in the region.

**Figure 1:**
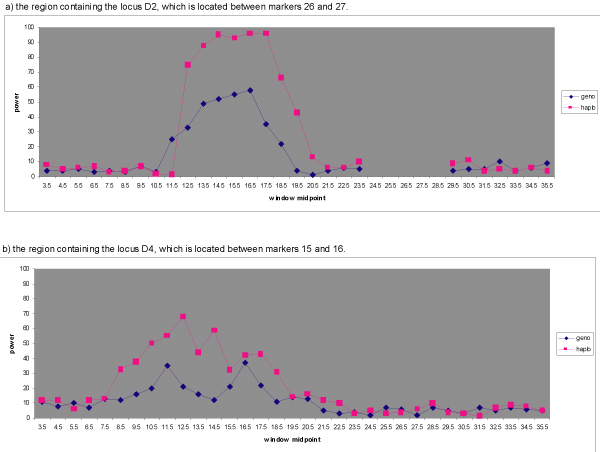
**Power of methods for Loci D2 and D4**. The power for each window is plotted at the midpoint of the window in terms of marker number within the region. The gap in the lines for D2 is for windows overlapping the end of the chromosome.

Figure 1 shows the power of the analysis for unphased and inferred haplotype data for individual windows in the region around D2 and D4. Both show very localized peaks, in windows overlapping the true site for D4, and greater power using inferred haplotypes than using unphased data. There are no windows overlapping D2.

## Discussion

Both methods have good power to detect the D2 locus in all four subpopulations. This is probably because D2 contributes to all three phenotypes used to identify affected individuals, but also due to the way the data was simulated for this locus, where the variant was inserted into haplotypes chosen for their similarity. This is exactly the scenario that the clustering approach is designed to exploit. Given the small sample size and the complex inheritance of the phenotypes, this is very encouraging. There is also a very localized peak, although it is a little way from the end of the chromosome. It is possible that this may be a result of LD in the region.

There is some power to detect D4, with the strongest signal in the Karangar subpopulation, which is also the only subpopulation in which there is any power to detect D3. This is again probably due to the phenotypes considered as affected in the different subpopulations. There is less power to detect D3 and D4 than for D2, probably because the haplotypes carrying the variant were chosen by their frequency rather than by their similarity. This does not fit the cladistic model, as haplotypes carrying the same mutation could be quite diverse. If the carrier haplotypes are very diverse, there is no advantage in cladistic analysis. There is also a more complicated relationship between phenotype and genotype for the phenotypes affected by D3 and D4, which may dilute the signal from any one locus.

D1 is not detected by either method in any of the four subpopulations. This is probably because the data was simulated with no LD between the disease locus and flanking markers. As the locus D1 itself was not genotyped, the lack of LD in the region makes it impossible to detect with association-based methods.

A small marginal effect was detected by the haplotype analysis for the modifier locus M1 in the Danacaa and Karangar populations. This is probably because it converts the phenotype P2 into P1. In the Danacaa sample individuals with P1 are 'affected' and individuals without P1 are not 'affected', so converting P2 into P1 will directly influence disease status. A similar effect occurs in the Karangar subpopulation. There is no marginal effect detected for the other modifier locus, M2, probably because it does not directly determine disease status.

## Conclusion

Our results suggest that a two-stage strategy of inferring haplotypes and analyzing the best-guess phase assignment for each individual appears to have more power than direct analysis of unphased multilocus genotypes. This is probably due to the smaller number of degrees of freedom in the test in the haplotype analysis. Haplotypes were simulated in different ways for the four disease loci, which affected the power of these methods to detect them. Cladistic-based analysis performs best when the haplotypes carrying a disease variant are similar, as simulated for locus D2, but has some success even when the disease site does not fit the cladistic model and the haplotypes do not necessarily fall into useful clades, as for loci D3 and D4. As expected, when there is no LD in the flanking region, there is no power to detect association with disease loci that are not themselves genotyped, as for locus D1.

A major disadvantage of the inferred haplotype analysis is the assumption that the inferred phase assignment is correct, which may result in biased estimates of LD between loci and inflated false-positive rates for association tests. Therefore, all possible phase assignments and corresponding probabilities for each individual should be included in the analysis [3-5]. Here there is little uncertainty in phase assignment, as shown by the correct false-positive rate for the test on the inferred data, but these results would not necessarily be expected to apply to regions with more uncertain haplotype resolution.

## Abbreviations

EM: Expectation maximization

GAW14: Genetic Analysis Workshop 14

LD: Linkage disequilibrium

SNP: Single-nucleotide polymorphism

## Authors' contributions

CD participated in the design of the study, performed the data analysis and drafted the manuscript. APM conceived of the study and participated in its design and coordination and helped to draft the manuscript.

## Figures and Tables

**Table 1:** False positive error rates for methods

	LD regions (95% CI)	No LD regions (95% CI)
Genotypes	0.056 (0.04, 0.072)	0.048 (0.033, 0.062)
Haplotypes	0.064 (0.046, 0.081)	0.054 (0.038, 0.069)

Mean false positive error rates for the two methods, in regions with and without LD, based on false-positive rates in four null regions. Averages were taken over all 100 replicate datasets over all four subpopulations.

**Table 2:** Power of methods for the disease Loci in the subpopulations

	Power [Accuracy]							
	Aipotu		Danacaa		Karangar		NYC	
				
	Geno^a^	Haplo^b^	Geno	Haplo	Geno	Haplo	Geno	Haplo

D1	0.06 [33.3]	0.05 [20]	0.06 [16.7]	0.07 [71.4]	0.04 [0]	0.06 [0]	0.07 [0]	0.07 [28.6]
D2	0.30	0.67	0.40	0.89	0.31	0.49	0.36	0.78
D3	0.05 [40]	0.07 [57.1]	0.06 [33.3]	0.04 [25]	0.14 [14.3]	0.17 [64.7]	0.04 [0]	0.09 [55.5]
D4	0.11 [36.4]	0.26 [46.2]	0.03 [0]	0.07 [14.3]	0.29 [31]	0.47 [63.8]	0.08 [37.5]	0.21 [42.9]
M1	0.01 [0]	0.06 [33.3]	0.07 [42.9]	0.16 [68.8]	0.01 [0]	0.14 [57.1]	0.06 [16.7]	0.06 [0]
M2	0.08 [12.5]	0.07 [14.3]	0.02 [0]	0.03 [0]	0.03 [0]	0.04 [0]	0.02 [50]	0.09 [11.1]

Power of unphased and inferred haplotype analyses to detect the four disease loci and two modifier loci in the four subpopulations.

^a ^Geno, unphased

^b ^Haplo, inferred

## References

[B1] Zondervan KT, Cardon LR (2004). The complex interplay among factors that influence allelic association. Nat Rev Genet.

[B2] Durrant C, Zondervan KT, Cardon LR, Hunt S, Deloukas P, Morris AP (2004). Linkage disequilibrium mapping via cladistic analysis of single-nucleotide polymorphism haplotypes. Am J Hum Genet.

[B3] Schaid DJ, Rowland CM, Tines DE, Jacobson RM, Poland GA (2002). Score tests for association between traits and haplotypes when linkage phase is ambiguous. Am J Hum Genet.

[B4] Stram DO, Pearce CL, Bretsky P, Freedman M, Hirschhorn JN, Altshuler D, Kolonel LN, Henderson BE, Thomas DC (2003). Modelling and E-M estimation of haplotype-specific relative risks from genotype data for a case-control study of unrelated individuals. Hum Hered.

[B5] Zaykin DV, Westfall PH, Young SS, Karnoub MA, Wagner MJ, Ehm MG (2002). Testing association of statistically inferred haplotypes with discrete and continuous traits in samples of unrelated individuals. Hum Hered.

